# Insight into impaired social functioning in dementia

**DOI:** 10.1017/S1041610223001783

**Published:** 2023-12

**Authors:** Andrew Sommerlad, Jessica Grothe, Sumiyo Umeda, Manabu Ikeda, Hideki Kanemoto, Gill Livingston, Melanie Luppa, Katherine P. Rankin, Steffi G. Riedel-Heller, Susanne Röhr, Maki Suzuki, Jonathan Huntley

**Affiliations:** 1 Division of Psychiatry, University College London, Gower Street, London WC1E 6BT, UK; 2 Camden and Islington NHS Foundation Trust, St Pancras Hospital, London, UK; 3 Institute of Social Medicine, Occupational Health and Public Health (ISAP), Medical Faculty, University of Leipzig, Germany; 4 Department of Psychiatry, Osaka University Graduate School of Medicine, Osaka, Japan. Department of Psychiatry, Daini Osaka Police Hospital, Osaka, Japan; 6 Department of Neurology, Memory and Aging Center, University of California, San Francisco, USA; 7 Global Brain Health Institute (GBHI), Trinity College Dublin, Dublin, Ireland; 8 School of Psychology, Massey University, Manawatu, New Zealand; 9 Department of Behavioral Neurology and Neuropsychiatry, Osaka University United Graduate School of Child Development, Osaka, Japan

## Abstract

**Background:**

People with dementia commonly have impaired social functioning and may not recognise this. This lack of insight may result in worse outcomes for the person and their family carers. We aimed to characterise insight into social functioning in dementia, and describe its association with dementia severity.

**Methods:**

Observational cross-sectional study of people aged >65 years with clinically diagnosed dementia and their family informants recruited from three sites in Germany, Japan and the United Kingdom. We used the Social Functioning in Dementia scale (SF-DEM), which assesses three domains: “spending time with other people” (domain 1), “communicating with other people” (domain 2), and “sensitivity to other people” (domain 3). We calculated lack of insight into social functioning as the discrepancy between the ratings of the participants with dementia and their informant. We described this discrepancy and the proportion of people with dementia whose rating was overestimated, congruent or underestimated compared to their family informant. We calculated the association between SF-DEM discrepancy score and total mini-mental status examination (MMSE) score and recall and attention/concentration subdomains.

**Results:**

In 108 participants with dementia (50.9% women), mean age = 78.9 (standard deviation, SD 6.5) years, and mean MMSE score = 22.7 (SD 3.7). Ratings of patients and informants for domain 1 did not differ, but patient-rating was higher than carer-rating for domain 2 (patient-rated score 11.2 (2.5), carer-rated score 10.1 (3.4); p = 0.003) and domain 3 (patient-rated score 9.7 (2.4), carer-rated score 8.1 (2.8); p < 0.001). Sixty (55.6%) people with dementia overestimated their overall social functioning, 30 (27.8%) underestimated, and 18 (16.7%) gave ratings congruent with their family informant. Performance on the MMSE, and its sub-domains was not associated with SF-DEM discrepancy score.

**Conclusions:**

We found that insight varies according to subdomains of social functioning, with people with dementia rating their communication and sensitivity differently, and usually higher than their carers. Researchers and clinicians should consider insight into social functioning in dementia as a multidimensional, rather than a unified, concept.

Clinicians should help family members understand and adapt by explaining their relative with dementia’s lack of insight about aspects of their social functioning.

